# A new species of *Charinus* Simon 1892 from Brazil, with notes on behavior (Amblypygi, Charinidae)

**DOI:** 10.3897/zookeys.621.9980

**Published:** 2016-10-03

**Authors:** Gustavo S. Miranda, Milena Milleri-Pinto, Thiago Gonçalves-Souza, Alessandro Ponce de Leão Giupponi, Nikolaj Scharff

**Affiliations:** 1 Center for Macroecology, Evolution and Climate, Natural History Museum of Denmark (Zoological Museum), University of Copenhagen, Universitetsparken 15, 2100 Copenhagen, Denmark, Copenhagen, Denmark; 2 Rua José Anchieta Fontana, 563/503, Edifício Letícia, Jardim 29090-400, Camburi, Vitória/ES, Brazil; 3 Laboratory of Phylogenetic and Functional Ecology (ECOFFUN), Department of Biology, Area of Ecology, Federal Rural University of Pernambuco (UFRPE), R. Dom Manoel de Medeiros s/n, Recife, PE, Zip Code 52171-900, Brazil; 4 Laboratório de Referência Nacional em Vetores das Riquetsioses, LIRN-FIOCRUZ, Rio de Janeiro, RJ, Brazil

**Keywords:** Activity rhythms, Atlantic Forest, behavioral repertory, taxonomy, Whip spider

## Abstract

A new species of *Charinus* is described and illustrated from the Brazilian Atlantic Forest. *Charinus ruschii* **sp. n.** is found in Santa Lúcia reserve, Espírito Santo state, and is sympatric with *Charinus brasilianus* and *Charinus montanus*. The new species can be easily distinguished from the other species of the genus by the combination of the following characters: number of spines on the pedipalp tarsus, size and shape of the female genitalia, shape of the sternum and number of teeth in the cheliceral claw. The behavioral repertory is also described for this species based on five hours of qualitative and 24 hours of quantitative observations in order to define the behavioral categories. Five behavioral categories were detected and 21 behavioral acts. The most conspicuous category was *Immobility*, followed by *Antenniform leg movement*, *Environmental exploration*, *Self-grooming*, and *Feeding*. It was also found that juveniles spend longer time inside the shelter, even during peaks of adult activity, which could be related to a survival strategy.

## Introduction

Whip spiders (order Amblypygi) are peculiar flattened arachnids with unique spiny, robust pedipalps and extremely elongate first pair of legs (Weygoldt 2000). The group is widely distributed in the tropics (Giupponi and Miranda (2016), Miranda et al. (2016); but see Miranda et al. (in press)), and consists of more than 200 species with the majority of the diversity in the family Charinidae Simon, 1892. Charinidae contains three genera, *Catageus* Thorell, 1889 with one species, *Charinus* Simon, 1892 with 70 species, and *Sarax* Simon, 1892 with 17 species. The genus *Charinus* has a pantropical distribution, occurring in the Caribbean region, Central and South America, Africa, Europe, Asia and Oceania, but most of the species have been described from the Americas. However, its diversity is still not completely revealed, as shown by the many new species discovered during the last decade (Armas 2006, 2010, Armas et al. 2016, Giupponi and Miranda 2016, Jocqué and Giupponi 2012, Miranda et al. in press, Miranda and Giupponi 2011, Miranda et al. 2016, Réveillion and Maquart 2015, Seiter et al. 2015, Teruel 2016, Teruel and Questel 2011, 2015, Torres-Contreras et al. 2015, Vasconcelos et al. 2016, Vasconcelos et al. 2013, 2014).

Like most other arachnids, whip spiders are nocturnal animals that hide in shelters during the day (such as rock crevices or inside and under logs) and venture out to forage at night (Pinto-da-Rocha et al. 2002, Weygoldt 2000). The biology of some Phrynidae and Phrynichidae species are known (e.g. reproduction, microhabitat selection/use, spatial orientation, and social behavior; reviewed in Weygoldt (2000) and Chapin and Hebets (2016)). However, little information is available for charinids, which counts with just few general behavioral studies examining reproductive biology and behavioral repertory (Gray and Robinson 1986, Pinto-da-Rocha et al. 2002, Weygoldt 2008).

Animal behavior is defined as any act performed by an animal and involves much more than movements for locomotion or displacement, it also encompass fine movements and inactivity (Del-Claro and Prezoto 2003). As a starting point to understand the biology and ecology of an animal, behavioral repertoires (or ethograms) are important tools and give information about animal’s main activities, peaks of activity, interactions, and how it distributes its activities during the day (Beck 1972, Del-Claro 2004, Lehner 1996). This kind of study allows quantitative comparisons of behavioral repertoires among different species and between individuals of different sexes (Osses et al. 2008). The observations of a given animal are represented in tables of qualification (description of behavioral categories) and quantification (frequency of each behavioral category in *t* time) of the acts (Del-Claro 2004, Santos et al. 2003).

Few studies have been published on the behavioral repertoires of arachnids, including Opiliones (Elpino-Campos et al. 2001, Osses et al. 2008, Pereira et al. 2004), Scorpiones (Mineo et al. 2003), Ricinulei (García et al. 2015), Schizomida (Oliveira and Ferreira 2014) and Thelyphonida (Punzo 2000). There are also few works on Amblypygi, including a study on *Charinus asturius* Pinto-da-Rocha, Machado and Weygoldt, 2002 (Pinto-da-Rocha et al. 2002), and two papers on *Heterophrynus* sp. (Beck 1972, Beck and Görke 1974).

This paper contributes to the knowledge of *Charinus* in the Neotropics with the description of *Charinus ruschii* sp. n. and its behavior repertoire. This species occurs in the Brazilian Atlantic Forest (state of Espírito Santo, Southeastern Brazil), and is the third *Charinus* species discovered in Santa Teresa municipality (Fig. 4). *Charinus ruschii* differs significantly from the other known species in the area (*Charinus brasilianus* Weygoldt, 1972 and *Charinus montanus* Weygoldt, 1972), as described in detail below.

## Materials and methods

Individuals of *Charinus ruschii* were collected at Estação Biológica de Santa Lúcia (EBSL), a biological station of 440 ha located 10 km from Santa Teresa (19°57'S; 40°31'W), in Espírito Santo state, southeastern Brazil (Mendes and Padovan (2000); Fig. 4). The EBSL is inside a remnant of Atlantic rainforest, in a geomorphologic crystalline complex with high rocky outcrops ranging in altitudes from 600 to 900 m (Thomaz 1996). Mean annual precipitation for Santa Teresa region is 1345 mm with a peak in November and a dry season in June and July (Mendes and Padovan 2000).

For nomenclature and measurements, the methods of Quintero (1981) are generally followed. The terminology of pedipalp and leg segments follows Harvey and West (1998). The article called tarsus by Harvey and West (1998) is divided here into tarsus and claw, as there is no fusion of these two segments in Charinidae. The spines of the pedipalpal patella and teeth of the chelicerae are counted from the apex to the base. Measurements of the entire type series were taken, and the measuring of the pedipalp articles was taken between the condyles of each segment in order to establish fixed points and comparable length. Total body length is usually used as diagnostic of *Charinus* species, but was not included here due to the contractile nature of the abdomen. Instead, the carapace width, pedipalp femur length, and femur I length seems to have more reliable diagnostic characters and are here used.

Photographs were made using a BK plus Imaging System from Visionary Digital (Palmyra, PA, USA; http://www.visionarydigital.com/) equipped with a Canon 7D digital camera at the Zoological Museum, University of Copenhagen
(ZMUC). Stacks of images from multiple focal planes were combined using Zerene Stacker (Zerene Systems LLC, http://zerenesystems.com/cms/stacker) and processed in Photoshop CS6 (Adobe, San Jose, CA, USA) to adjust color, brightness, and contrast, and remove blemishes. The plates were mounted in Corel Draw X5 (Corel, Mountain View, CA). One female was examined with scanning electron microscopy (SEM). The SEM work was carried out with a JEOL JSM-6390LV at the Center for Scanning Electron Microscopy of the Museu Nacional/UFRJ.

The distribution map was produced and edited using ArcGis 10.2 (ESRI 2014) with vector layers for countries and states, and a raster background layer for the vegetal cover and urban occupation (made available by the Brazilian Ministry of the Environment, http://mapas.mma.gov.br/mapas/aplic/probio/datadownload.htm). The coordinates were obtained with Google Earth given the localities in the labels and information in the literature.

Abbreviations of the repositories cited:

MNRJ
 Museu Nacional do Rio de Janeiro, Rio de Janeiro, Brazil 

MZSP
 Museu de Zoologia da Universidade de São Paulo, São Paulo 

SMF
 Senckenberg Naturmuseum und Forschungsinstitut, Frankfurt, Germany 

The behavioral repertory of *Charinus ruschii* was recorded in the Laboratory of Arachnology at the *Escola Superior São Francisco de Assis/ESFA*, Brazil. Between June and July 2004, 17 specimens of *Charinus ruschii* (12 adult females and 5 juveniles) were collected inside caves and rock crevices at the study area. The individuals were kept separately from each other in captivity in plastic boxes (15 cm long × 11 cm wide × 7 cm high), which contained one rock (that was used by them as a shelter), mud as substrate, and a piece of wet cotton to maintain humidity. Each plastic box received a number that was used in the randomization process (see below). The laboratory room was kept under darkness as this species lives in caves and rock crevices, and red light was used during observations to avoid disturbing the individuals. The whip spiders were fed with fruit flies (*Drosophila* sp.), moth flies (*Psychoda* sp.), and unidentified species of butterflies and moths every three days (one prey per day of feeding). Five hours of observations allowed the definition of five categories and 21 different behavioral acts (Table 1). The individuals were kept seven days under captivity before the behavioral observations.

**Table 1.** Category description and behavioral act frequencies of *Charinus ruschii* sp. n. in 1440 minutes of observation.

Categories/behavioral acts	Frequency (%)
**Immobility: stand still in an environment**	**48.89**
Stand still with abdomen leaning over the ground: specimen stays totally still outside the shelter with the body leaning on the ground	6.81
Stand still with abdomen distant from the ground: specimen stays totally still outside of the shelter with abdomen distant from the ground	20.00
Inside the shelter: specimen stays still inside the shelter with the body leaning over the ground	22.08
**Antenniform leg movement: stand still with the antenniform legs in movement**	**41.60**
Stand still with abdomen leaning over the ground and moving antenniform legs backward: specimen stays still and smoothly moving antenniform legs backward	2.92
Stand still with abdomen distant from the ground and antenniform legs waving under the body: specimen stays still with alternate movements of the antenniform legs backward and forward	31.25
Stand still with antenniform legs touching the ground: specimen stays still and touching the ground with antenniform legs	1.53
Stand still with antenniform legs erect: keep the body distant from the ground and smooth movements of the antenniform legs over the body	1.94
Stand still with antenniform legs pointing forward: antenniform legs in a forward position making smooth movements	3.96
**Environmental exploration**	**6.11**
Laterally walking: walking to the sides inside the plastic box, like a crab	1.18
Walking with the antenniform leg erect and pointed forward: slowly walking without moving the antenniform legs and in a forward position	2.36
Walking with antenniform legs erect and pointed backward: slowly walking without moving the antenniform legs and in a backward position	0.14
Walking and touching the ground with antenniform legs: slowly walking touching and feeling the ground with antenniform legs	0.56
Walking with antenniform legs pointed forward and second pair of legs touching the ground: slowly walking throughout the box with antenniform legs in a forward position and second pair of legs touching the ground	0.55
Walking with first and second pair of legs touching the ground: slowly walking all over the box touching its walls with antenniform legs and the ground with second pair of legs	1.04
Walking with antenniform legs in a lateral position and second pair of legs touching the ground: walking with antenniform legs in a perpendicular position in respect to body axis and second pair of legs touching the ground	0.28
**Feeding**	**0.76**
Eating a prey: to lacerate the prey with the chelicerae, with abdomen distant from the ground.	0.76
**Self-grooming**	**2.64**
Cleaning the pedipalps: rub the pedipalps all over the chelicerae	1.18
Cleaning first pair of legs: rub the antenniform legs over the pedipalps and/or the chelicerae	0.69
Cleaning second pair of legs: rub the second pair of legs over the pedipalps and/or the chelicerae	0.35
Cleaning third pair of legs: rub the third pair of legs over the chelicerae	0.07
Cleaning fourth pair of legs: rub the fourth pair of legs over the chelicerae	0.35

Twenty-four hours of quantitative observations were made for 24 days, in which 60 observations of 1 minute was performed each day. The hour of the day to be studied was randomly chosen (24 pre-defined days) and thus was spread throughout the whole study. That is, in day 1 we could perform the study from 10 am to 11 am, whereas in day 2 from 7 pm to 8 pm, and so on. The specimens were numbered from 1 to 17 and the order to be observed was randomly defined before the behavioral study (following Pinto-da-Rocha et al. (2002); Machado, G. (pers. comm, 6 April 2004)). In total, 1440 minutes of observation were recorded totaling 24 hours and covering day and night. During the day, and after feeding, whip spider specimens remained resting. Voucher material is deposited in the Arachnology section of the Museu Nacional do Rio de Janeiro, Universidade Federal do Rio de Janeiro
(MNRJ). The voucher numbers are in the list of type material.

### Additional material examined

*Charinus acaraje* Pinto da Rocha, Machado & Weygoldt, 2002: Brazil, Bahia, Santa Luzia, Gruta do Lapão, R.L.C. Baptista *leg.* (3 males, 5 females, 1 juvenile, MNRJ 9297).

*Charinus asturius* Pinto da Rocha, Machado & Weygoldt, 2002: **holotype**: Brazil, São Paulo, Ilha Bela, Morro Pacuíba [23°44'S 45°19'W], i.1998, G. Machado *leg.* (1 male, MZSP 18930); **paratypes**: same data as holotype (1 female, MZSP 18930; 1 female with empty egg sac and numerous prenymphal exuviae, MZSP 18934); same locality as holotype, 18.i.1999, R. Pinto-da-Rocha and G. Machado *leg.* (4 males, 3 females, 1 immature female, 6 protonymphs, MZSP 16900).

*Charinus brasilianus* Weygoldt, 1972: **holotype** and **paratype**: Brazil, Espírito Santo, Serra, 10 km north of Vitória, 200–400 m, xi.1970, P. Weygoldt *leg.* (1 male, 1 female, MNRJ 9014); **other paratypes**: same data as holotype (3 males, 3 females, 3 juveniles, SMF 25397); **additional material**: Nova Valssugana [19°54'09"S 40°39'31"W], Santa Teresa, v.2005, T. Gonçalves-Souza *leg.*, G.S. Miranda det. (2 males, MNRJ 9271); Reserva Santa Lúcia, Santa Teresa, 15–19.x.2003, Almeida, Baptista, Giupponi, Mendes and Pedroso *leg.*, G.S. Miranda det. (1 female, MNRJ 9232).

*Charinus eleonorae* Baptista & Giupponi, 2003: **paratypes**: Brazil, Minas Gerais, Itacarambi, Gruta Olhos d’Água, 26.vi.2001, R.L.C. Baptista & A.P.L. Giupponi *leg.* (7 males, 6 females, 2 males juvenile, 2 females juvenile, 2 small juveniles, MNRJ 9033).

*Charinus insularis* Banks, 1902: Ecuador, Galapagos Islands, Isla Santa Cruz, Turtle Bay, 14.i.1965, W.D. Stockton det. (1 male, 1 female, Royal Belgian Institute of Natural Sciences); Ferme Horuerneru, sous de pierres dores en ravim argilex, alt 250 m, ix.1964, J. and N. Leleup *leg.*, W.D. Stockton det. (2 females, Royal Belgian Institute of Natural Sciences).

*Charinus jibaossu* Vasconcelos, Giupponi & Ferreira et al., 2014, **paratypes**: Brazil, Minas Gerais, Pains, Gruta da Vila Corumbá [20°19'55.8"S 45°36'43.23"W], 25.i.2009, R.A. Zampaulo *leg.* (1 male, MNRJ 9217); Gruta da Mineração, 25.i.2009 (1 female, MNRJ 9277).

*Charinus koepckei* Weygoldt, 1972: **holotype**: Peru: Am Weg v. Chala n. Chaparra (Küste; lichte Waldloma, u.Stein.) um 500 m (1 female, SMF 25762).

*Charinus montanus* Weygoldt, 1972: **holotype** and **paratype**: Brazil, Espírito Santo, Domingos Martins, 1000 m, xi.1970, P. Weygoldt *leg.* (1 male, 1 female, MNRJ 9015); **other paratypes**: Domingos Martins, 50 Km. W. Vitória. Bergwäld. a., xi.1967, P. Weygoldt and Helversen *leg.* (5 females, 5 males, 3 juveniles, SMF 25398); **additional material**: Santa Teresa, M. Milleri-Pinto and T. Gonçalves-Souza *leg.*, G.S. Miranda det. (4 females, 1 male, MNRJ 9243); Santa Teresa, Estação Biológica Santa Lúcia, G.S. Miranda det. (2 males, 2 females, MNRJ 9273); Santa Teresa, Reserva Santa Lúcia, 15–19.x.2003, Almeida, Baptista, Giupponi, Mendes & Pedroso *leg.*, A.P.L. Giupponi det. (5 males, 3 juveniles, MNRJ 9087).

*Charinus mysticus* Giupponi & Kury, 2002: **paratype**: Brazil, Caverna Encantados, Gentil do Ouro, 16 km from Santo Inácio, road to Gameleira; cave with stream, about 8 m from entrance, 28.ix.1991, G. Skuk *leg.* (1 female, MNRJ 9022).

*Charinus potiguar* Vasconcelos Giupponi & Ferreira, 2013: **paratypes**: Brazil, Rio Grande do Norte, Caverna do Pau, Felipe Guerra [5°35'34.19"S 37°41'14.64"W], 08.i.2007, R.L. Ferreira *leg.* (1 male, MNRJ 9212); Caverna do Geilson [05°35'53.23"S 37°41'17.56"W], 23.iv.2007, D. M. Bento *leg.* (1 female, MNRJ 9213).

*Charinus troglobius* Baptista & Giupponi, 2002: **paratype**: Brazil, Bahia, Carinhanha, Gruna do Zé Bastos, Serra do Ramalho, 28.vi.2001, R.L.C. Baptista & A.P.L. Giupponi *leg.* (2 males, MNRJ 9069, 9081; 1 female, MNRJ 9078).

## Results

### Taxonomy Family CHARINIDAE Quintero, 1986 Genus *Charinus* Simon, 1892

#### Charinus ruschii sp. n.

Taxon classificationAnimaliaAmblypygiCharinidae

http://zoobank.org/1AAB23A6-5DD9-4580-A97B-956F2BF0E000

**Figure 1.**
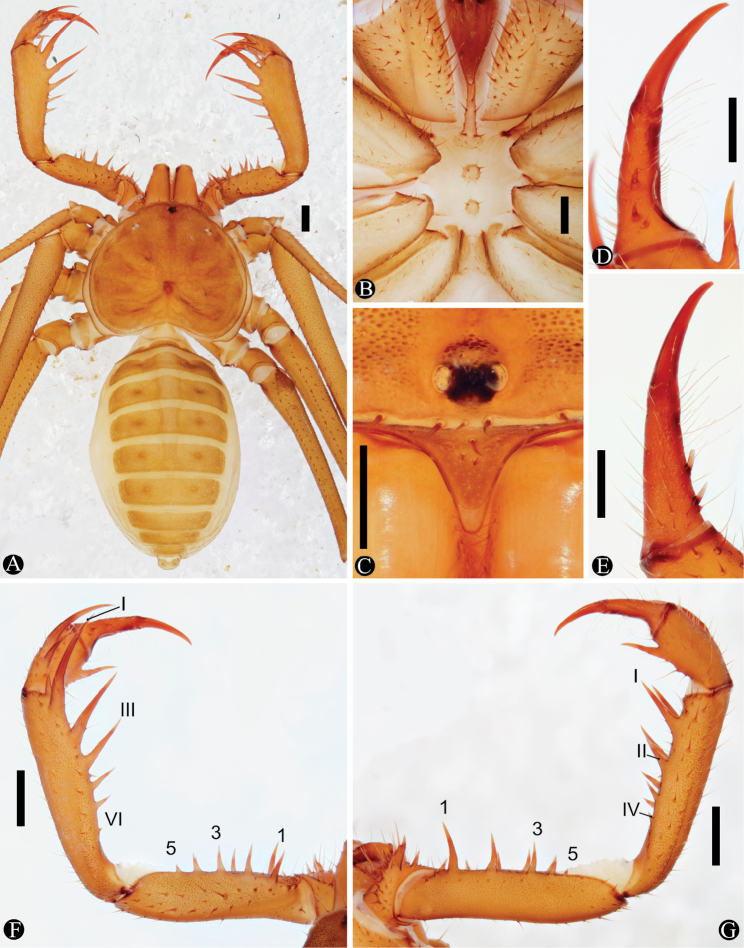
Habitus and details of *Charinus ruschii* sp. n. (holotype, MNRJ 9235). **A** Habitus dorsal **B** Sternum **C** Frontal process **D** Dorsal view of left pedipalp tarsus and claw **E** Frontal view of left pedipalp tarsus and claw **F** Dorsal view of left pedipalp **G** Ventral view of left pedipalp. Scale bars: **A, F, G**: 1 mm; **B, C, D, E**: 0.5 mm.

**Figure 2.**
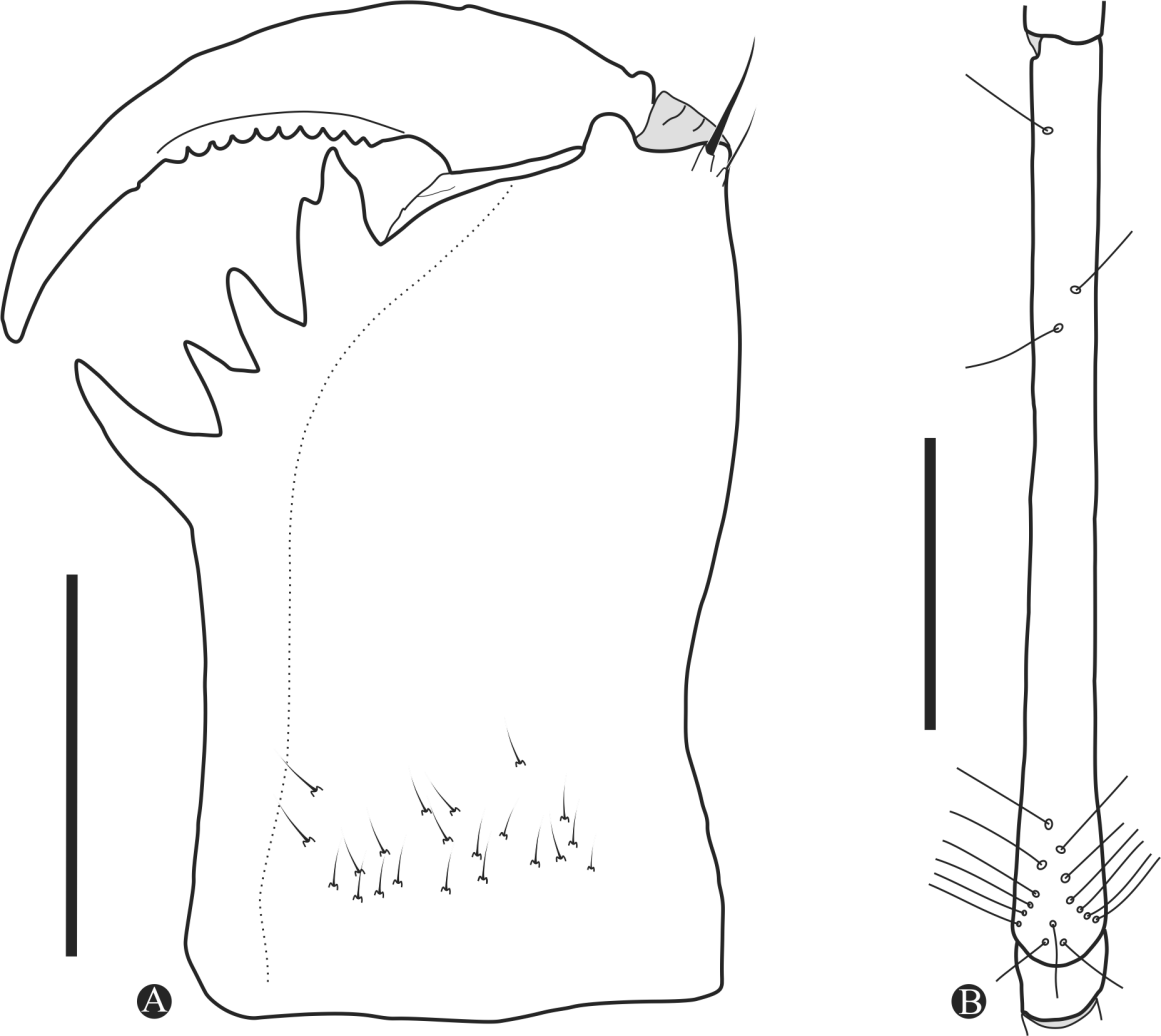
Chelicerae (**A**) and distitibia of leg IV (**B**) of *Charinus ruschii* sp. n. (MNRJ 9237 and MNRJ 9235, respectively). Scale bars: 1 mm

**Figure 3.**
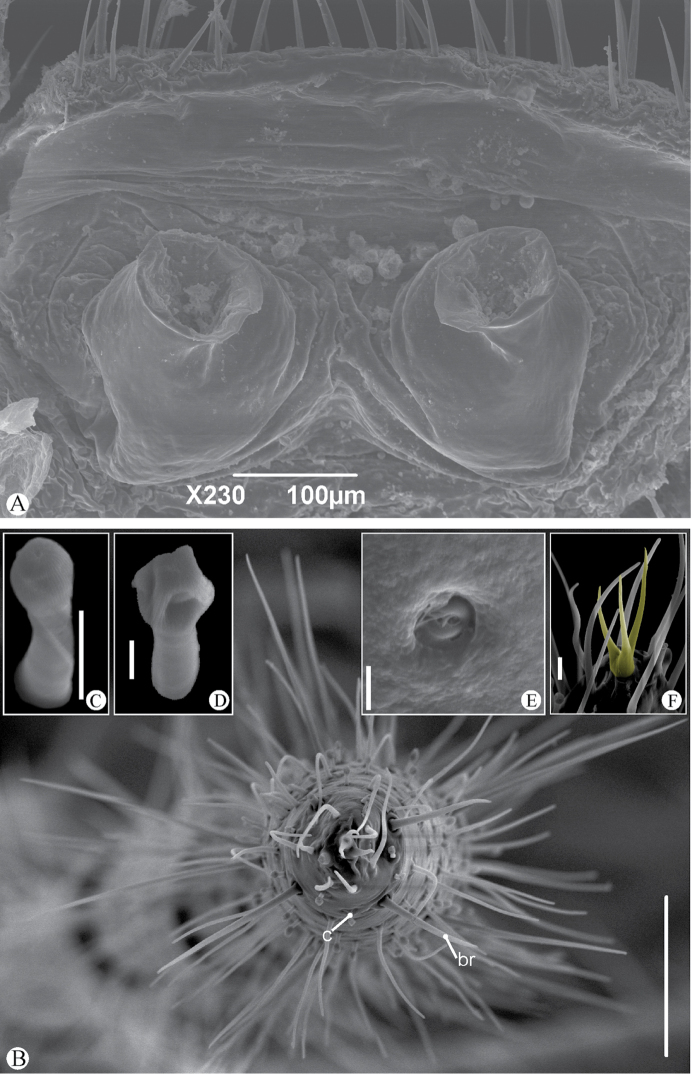
*Charinus ruschii* sp. n. female gonopod and detail of leg I (MNRJ9237). **A** Dorsal view of the female gonopod **B** Frontal view of the tip of leg I **C** Detail of sensitive hair type 1 **D** Detail of sensitive hair type 2 **E** Glandular opening close to the tip of the tarsus **F** Modified claw in the tip of the leg. Abbreviations: br: bristle sensilla; c: club sensilla. Scale bars: **B**: 100 um; **C**: 5 um; **D, E**: 2 um; **F**: 10 um.

**Figure 4.**
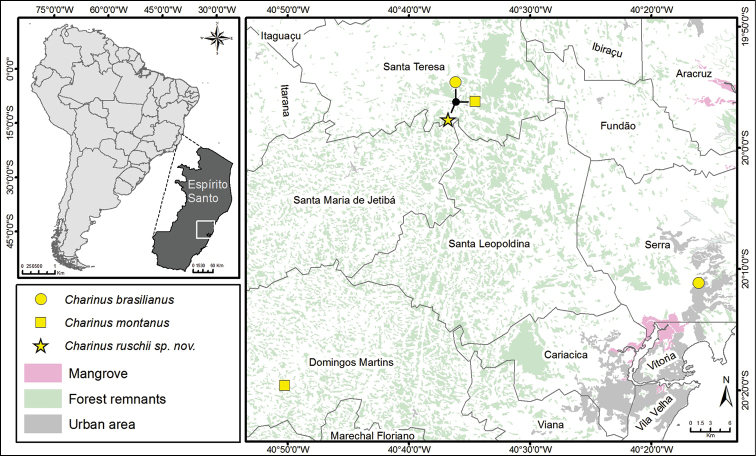
Distribution map of *Charinus* in the state of Espírito Santo.

Figures 1 , 2 , 3 , 4

##### Diagnosis.

*Charinus ruschii* can be easily recognized by the sucker-like female gonopod, presence of three spines on the pedipalp tarsus, large size (carapace *circa* 80% wider than that of *Charinus brasilianus* and *Charinus montanus*), small unique platelets of the sternum, similar size of the proximal segment of tarsus I compared to the other segments, and cheliceral claw with 10 teeth.

##### Type material.

**Holotype**: Brazil, Espírito Santo, Santa Teresa municipality [19°56'12.60"S 40°35'53.99"W], T. Gonçalves-Souza and M. Milleri-Pinto *leg.* (1 female, MNRJ 9235). **Paratypes**: same data as holotype, T. Gonçalves-Souza and Milena *leg.* (4 females, 1 juvenile, MNRJ 9237); same data as holotype, T. Gonçalves-Souza and Milena *leg.* (2 females, MNRJ 9235); Estação Biológica Santa Lúcia, v.2005, T. Gonçalves-Souza *leg.* (1 female, MNRJ 9272); Man. Livre Div. Santa Teresa, M. Milleri-Pinto and T. Gonçalves-Souza *leg.* (2 females, MNRJ 9303).

##### Etymology.

The species is named after the late agronomist and naturalist Augusto Ruschi (1916-1986), who played an important role in the investigation and conservation of the Atlantic Forest, and who was born in the city of the type locality of the new species. He was also involved in the creation of *Estação Biológica Santa Lúcia* (Biological Station *Santa Lúcia*, a forested reserve) where the new species was found. In 2016 is also the centenary of his birth.

##### Description.

***Carapace*** (Fig. 1A): flattened, wider than long (1.4 times), slightly bent downwards below lateral eyes; thin median furrow reaching fovea starting from median eye tubercle. Anterior margin rounded, with 6 frontal setae. Frontal process large, triangular, not visible from above. Three pairs of shallow furrows on lateral side of carapace, and an oval fovea. 1st pair of furrows placed just behind the lateral boss; furrows not reaching the middle line. Median eyes and tubercle present. Lateral eyes well developed, 1 small setae behind each triad of eyes; lenses directed upwards and anteriorly.

***Sternum*** (Fig. 1B): 4-segmented, all articles well sclerotized. Tritosternum with round basis, projecting anteriorly in a small blunt tubercle, surpassing the base of the pedipalp coxae, with 2 apical, 2 median and 2 basal setae, with smaller ones spread from the middle to the base. Middle piece (tetrasternum) in one convex piece, with pair of large setae in its apex, and several small ones at its base. Third piece (pentasternum) formed by 1 convex piece, smaller than middle piece, with 2 long setae at its top and several setae at its base. Sternites separated from each other by the length of the third piece. Metasternum paired in its anterior half, with an anterior setae in the membranous region followed by 2 to 3 setae in the sclerotized area, in a longitudinal row from the non-sclerotized to the sclerotized region; distal border with a small elevation bearing 6–8 large setae.

***Abdomen*** (Fig. 1A): oblong, with almost indistinguishable punctuations. Ventral sacs not present.

***Chelicera*** (Fig. 2A): cheliceral furrow with 4 internal (prolateral) teeth; first tooth (upper) bifid with proximal cusp much larger than distal cusp. Third tooth slightly thinner and shorter than second. Fourth tooth one third larger than the third. No tooth in the external row of the basal segment. Mesal face with several small setae. Claw with 10 denticles.

***Pedipalp*:** **Trochanter** (Fig. 1G): large spiniform ventral apophysis, located in the posterior border of the trochanter, bearing 13 large setae, and with a blunt tip pointed forward; 2 subequal spines, one at about the center of the anterior row of setiferous tubercles, the other at the external border, above the apophysis, slightly curved inwards. ***Femur*** (Fig. 1F, G): 5 to 6 dorsal spines (I>II>III>IV>V>VI) with 2 prominent setiferous tubercles before first spine; 5 ventral spines (I>II>III>IV>V), with 1 small spine displaced from the main series, dorsal to spine 1; with 2 small setiferous tubercles before first spine. ***Patella*** (Fig. 1F, G): 6 dorsal spines (I>II>III>IV>V>VI); one setiferous tubercle distal to I (about one third the size of I); spine I with 3 large and several small setae in the first third; spine II with 3–4 large setae at basal third; spine III with 1 setae in its distal third. 4–5 ventral spines decreasing in size. ***Tibia*** (Fig. 1F, G): 2 dorsal spines, the basal 2/3 the size of the distal. One ventral spine at distal half, 2/3 the basal spine dorsal. ***Tarsus*** (Fig. 1D, E): 3 dorsal spines (present since early stages of development); 2 distal, subequal between each other and 1/5 the size of the article; the proximal spine small, circa of 1/3 the size of the other two, positioned close to the proximal spine and with long setae in its base. Cleaning organ about 1/2 of the article length. ***Claw*** (Fig. 1D, E): long, with an acute, curved tip.

***Legs*:** all segments setose. Ventral corner of the prolateral face of femora II–IV projecting in a distinct spiniform process. Femur length: I>III>II>IV. Tibia I with 23 articles; distal segments with 2 small trichobothria, 1 on the dorsal and 1 on the lateral (mesal) side of the segment; 1 trichobothria in the second, third and fourth (from distal to proximal) segments, close to the distal border, all dorsally positioned; no trichobothria on other segments. Tarsus (basitarsus+distitarsus) I with 41 articles covered with large number of sensilla (Fig. 3B–F). Tip of leg I with small modified claw, emerging from common base (Fig. 3F). Lateral claws smaller than middle claw. Segments covered with at least 2 sensilla types, the bristle sensilla (*br*) and the club sensilla (*c*; Figs 3C, D). The club sensilla are found in the first 3 or 4 segments of tarsus I, whereas the bristle sensilla are present in all segments of tarsus and tibia I, decreasing in number from the tip to the base of the segments.

***Leg IV*:** ***Basitibia***: divided into 4 pseudo-articles, with 1 trichobothrium on the first third of the last pseudo-segments (trichobothrium *bt*). ***Distitibia*** (Fig. 2B): 3 proximal and 15 distal trichobothria (total of 18); trichobothrium *bc* closer to *sbf* than to *bf*; *sf* and *sc* with 6 trichobothria. Basitibia-distitibia length DT>BT1>BT4>BT3>BT2. Tarsus: with well marked white ring in the distal part of first segment of distitarsus IV.

##### Color pattern

**(alcohol preserved material)**. Chelicerae, pedipalps, carapace, and abdomen yellowish-brown; tibia and tarsus of legs lighter colored. Live animals grey.

##### Genitalia.

female gonopod (Fig. 3A): posterior margin of genital operculum straight, with several setae along its margin and on its surface. Gonopods sucker-like, with a broad base, a constriction in the middle of the short stalk, and a rounded opening of the atrium; stalk slightly curved inwards. Base soft and wrinkled.

##### Male.

not known.

### Behavioral repertory

The most conspicuous behavioral category was *Immobility* (48.89%), followed by *Antenniform movement* (41.60%), *Environmental exploration* (6.11%), *Self-grooming* (2.64%) and *Feeding* (0.76%) (Table 1). Within the *Immobility* category, the most frequently performed behavioral act was *Inside the shelter* (22.08%), and 83.04% of the total were performed by juvenile. In the *Antenniform movement* category the behavioral act *Stand with the abdomen distant from the ground and first pair of legs waving over the body* was the most frequent (31.25%), showing the relevance of the antenniform legs and its sensory structures (Beck et al. 1977, Hebets 2002, Hebets and Chapman 2000, Igelmund 1987), which main functions are: (1) intra-specific signals, (2) navigation, (3) olfactory sensitivity, and (4) foraging. The main act of *Environmental exploration* was *Walking with first pair of legs erect and pointed forward* (2.36%); due to sensorial function, the forward position of the first pair of legs could be related to foraging (Hebets 2002, Weygoldt 2000). Likewise, Pereira et al. (2004) found better significance in environmental exploration categories linked with the use of the second pair of legs in the harvestman *Ilhaia cuspidata* Roewer, 1913, that has sensorial function too. In the *Self-grooming* category, *Cleaning the pedipalps* was the most expressive act (1.32%). Whip spiders in general use their chelicerae and pedipalps for cleaning their appendages (Alexander 1962, Weygoldt 2000); in the tarsus of the pedipalps is present a structure formed by two parallel rows of modified setae that cleans the legs (i.e. cleaning organs or cleaning brush, *sensu* Weygoldt (2000)). Legs with higher percentage of cleaning were the antenniform legs (0.69%), probably related to their sensorial function (i.e. sensilla and trichobothria; Foelix et al. (1975); Fig. 3). Also, Pereira et al. (2004) observed that legs with sensorial structures were the most cleaned in *Ilhaia cuspidata*. *Feeding* represented only 0.76% of all behaviors. Specimens of *Psychoda* sp. were always eaten when given to the whip spiders, whereas drosophila and butterflies were found dead after three days of being offered. The motivation of *Charinus ruschii* to feed on live animals was similar to that of *Damon gracilis* Weygoldt, 1998 (Weygoldt 2000), and different to that of *Heterophrynus batesii* (Butler, 1873) which accept dead animals (Beck and Görke 1974).

**Figure 5.**
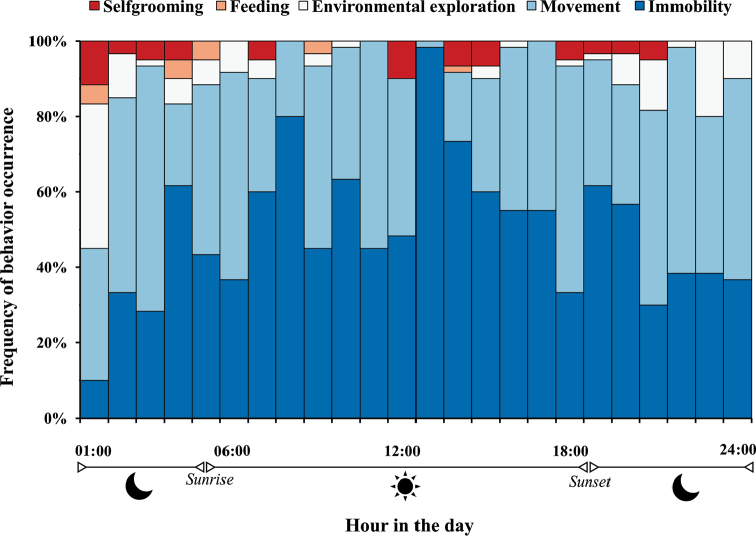
Behavioral category frequency for all *Charinus ruschii* sp. n. individuals in 1440 minutes of observation. Sun icon represents the diurnal period and moon icon the dark period.

Similarly to other whip spiders and other arachnids such as harvestmen, scorpions, spiders, and vinegaroons (e.g., Foelix (1996); Punzo (2000); Elpino-Campos et al. (2001); Pinto-da-Rocha et al. (2002); Mineo et al. (2003)), *Charinus ruschii* shows highest peaks of immobility during the day than the night, beginning its period of activity (*Environmental exploration*) at dusk (i.e. 18:00 hrs). The *Immobility* behavior was common from 08:00hrs to 14:00 hrs, and *Environmental exploration* was between 22:00hrs and 02:00hrs. Despite of the described patterns of immobility and foraging, some *Charinus ruschii* specimens were inactive during the night and feed themselves during the day (Fig. 5), which could be explained by the lower or non-incidence of light during the day in the caves where they live. Day activities were also observed in other cave species, such as *Charinus asturius* and two genera of harvestmen, *Iporangaia* Mello-Leitão, 1935 and *Iguapeia* Mello-Leitão, 1935 (Hoenen and Gnaspini 1999).

### Remaining inside the shelter

In the laboratory experiment, specimens of *Charinus ruschii* remained outside the shelter during the day, as observed by Pinto-da-Rocha et al. (2002) in *Charinus asturius*. This is different to observations *in situ* for both *Charinus ruschii* and *Charinus asturius*.

**Figure 6.**
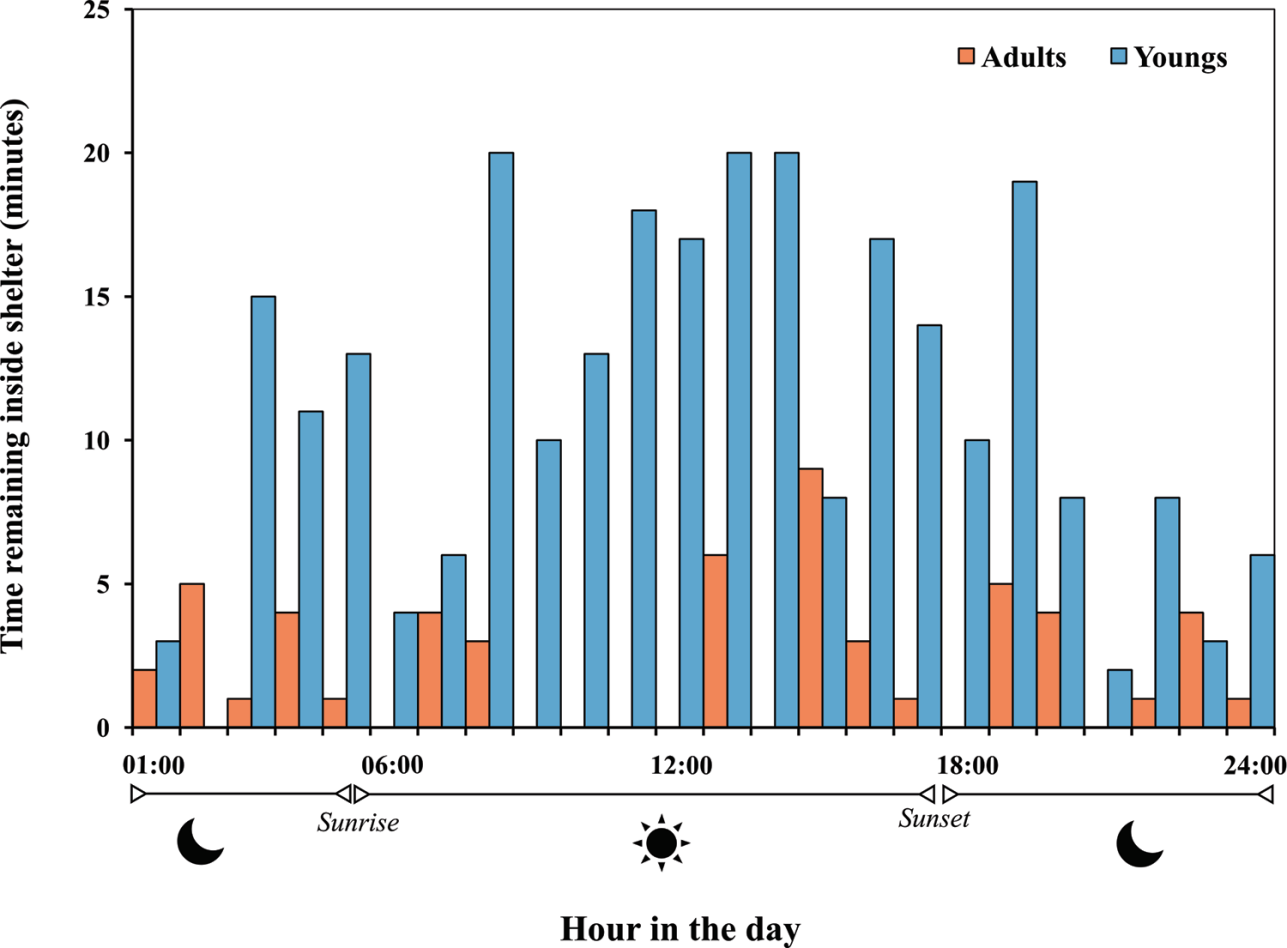
Difference between time remaining inside shelters in juveniles (n = 5) and adults (n = 12) amblypygids.

In average, each adult (n = 12) spent 4 minutes of their total time inside the shelter (i.e. 1440 minutes), while the mean for juveniles (n = 5) was 13.2 times higher than adults (mean = 52.8 min; test w for separate variance estimates *t* = 7.875, *P* < 0.0001; Fig. 5). Field observations of juvenile *Charinus montanus* show high rates of recapture in their shelter (i.e. high site fidelity). These data suggest that juveniles probably spend more time inside the shelter and this can be considered a survival strategy, which decreases the chances of being caught during the foraging periods (Chapin and Hebets 2016); the time spent foraging is also smaller than those spent by the adults, the juveniles adopt a “sit-and-wait” hunting strategy. Even in the peaks of activity (22:00 hrs and 02:00 hrs; Figs 5 and 6), most juveniles stayed inside the shelter. Salvestrini and Gasnier (2001) studied the differences in activity patterns between juveniles and adults of females and males of the spiders *Ctenus amphora* Mello-Leitão, 1930 and *Ctenus crulsi* Mello-Leitão, 1930, and confirmed that juvenile spiders were significantly more sedentary than adults from both sexes in *Ctenus amphora* and male adults from *Ctenus crulsi*. They suggested that sedentary behavior could be related to a foraging strategy, diet and resource available. Even more, Kreiter and Wise (1996), Schmitt et al. (1990) and Sullivan and Morse (2004) argued that, in general, adult spiders show more activity than juveniles, and that the difference between males and females is related to a sexual behavior in searching for males.

## Discussion

### Taxonomy

*Charinus ruschii* has a sucker-like female gonopod, which associates it with the *Charinus brasilianus* species group (*sensu* Weygoldt (2005)). The *brasilianus* species group occurs in the eastern part of South America and Oman, and includes *Charinus asturius*, *Charinus acaraje*, *Charinus brasilianus*, *Charinus bromeliaea* Jocqué and Giupponi, 2012, *Charinus caatingae* Vasconcelos & Ferreira, 2016, *Charinus eleonorae*, *C iuiu* Vasconcelos & Ferreira, 2016, *Charinus jibaossu*, *Charinus montanus*, *Charinus mysticus*, *Charinus potiguar*, *Charinus taboa* Vasconcelos, Giupponi & Ferreira, 2016, and *Charinus troglobius* from South America, and *Charinus dhofarensis* Weygoldt, Pohl & Polak, 2002 from Oman.

*Charinus ruschii* is a rather large species (see Table 2), but not as large as *Charinus mysticus* and *Charinus jibaossu*, the two largest species of the group. *Charinus mysticus* and the new species also have three spines on the pedipalp tarsus, trichobothria *bc* closer to *sbf* than to *bf*, and short stalk of the female gonopod. However, *Charinus mysticus* can be easily distinguished from the new species by the development of the median eyes and tubercle (reduced in *Charinus mysticus* and well-developed in *Charinus ruschii*), the shape of the frontal process apex (rhombus in the old species and acute in the new), and the number of teeth on the chelicerae claw (4 in *Charinus mysticus* and 10 in *Charinus ruschii*).

**Table 2.** Measurements and means sizes (in mm) of the three sympatric species from Santa Teresa (ES, Brazil): *Charinus ruschii* sp. n., *Charinus brasilianus* and *Charinus montanus*.

Species	Sex	Carapace length	Carapace width	Pedipalp femur length	Pedipalp patella length	Pedipalp tibia length	Pedipalp tarsus length	Pedipalp claw length	Femur I	Tibia I	Tarsus I
*Charinus ruschii* sp. n.	F	3.40	4.85	2.81	2.72	1.31	2.00	0.79			
	F	3.56	5.06	3.19	3.13	1.50	1.04	0.92	10.90	19.70	20.30
	F	3.50	4.90	2.80	2.68	1.36	1.00	0.83	9.23	28.70	28.20
	F	3.75	5.28	3.60	3.50	1.65	1.20	1.04	11.41	18.90	20.00
	F	3.44	4.88	3.50	3.00	1.50	1.13	0.94	10.90	18.40	19.00
	F	3.40	4.70	2.90	2.75	1.40	1.00	0.80	9.10	15.50	15.90
	F	3.52	5.20	3.15	3.05	1.50	1.10	0.90	10.51	17.80	18.90
	F	4.7	6.50	5.25	5.00	1.88	1.31	1.25	12.90	23.40	
	F	4.49	6.41	5.44	5.19	1.88	1.56	0.63	14.20	27.10	27.20
**Mean**		**3.75**	**5.31**	**3.63**	**3.45**	**1.55**	**1.26**	**0.90**	**11.14**	**21.19**	**21.36**
*Charinus brasilianus*	F	3.00	4.50	2.55	2.45	1.15	0.84	0.72	6.80	10.90	11.28
	M	3.05	4.45	3.05	3.10	1.20	0.80	0.60			
	M	3.68	5.28	7.50	7.50	1.60	0.88	0.90	7.95		
	M	2.88	5.19	5.69	5.94	1.44	1.00	0.65	7.69	13.85	11.54
	M	3.44	5.52	6.67	6.79	1.80	1.10	0.80	8.97	16.54	15.64
**Mean of females**		**3.00**	**4.50**	**2.55**	**2.45**	**1.15**	**0.84**	**0.72**	**6.80**	**10.90**	**11.28**
**Mean of males**		**3.26**	**5.11**	**5.73**	**5.83**	**1.51**	**0.95**	**0.74**	**8.21**	**15.19**	**13.59**
*Charinus montanus*	F	3.00	4.81	2.35	2.50	1.20	0.75	0.75	5.94	10.64	7.31
	F	2.80	4.45	2.00	2.03	1.00	0.66	0.63	5.40	10.00	7.31
	F	2.50	4.10	1.72	1.72	0.92	0.64	0.58	4.94	9.10	6.03
	F	2.05	4.00	1.75	1.63	0.97	0.60	0.60	4.81	8.46	5.77
	F	2.55	4.15	1.88	1.72	0.94	0.69	0.78	4.70	9.36	6.79
	M	3.10	4.90	3.60	3.60	1.15	0.75	0.65	6.64	10.90	8.21
	M	2.94	4.50	3.25	3.25	2.19	1.81	0.63	5.64	9.62	
	M	3.30	4.95	3.85	**4.10**	1.20	0.80	0.75	6.28	11.54	
	M	2.95	4.65	3.56	3.75	1.94	1.25	1.06	6.00	9.62	5.77
	M	2.61	4.69	2.94	2.88	1.10	0.72	0.68	5.84	9.74	7.05
**Mean of females**		**2.58**	**4.30**	**1.94**	**1.92**	**1.01**	**0.67**	**0.67**	**5.16**	**9.51**	**6.64**
**Mean of males**		**2.98**	**4.74**	**3.44**	**3.52**	**1.52**	**1.07**	**0.75**	**6.08**	**10.28**	**7.01**

*Charinus jibaossu* is quite similar to the new species, despite its larger size, but differences occur in the number of ventral spines on the pedipalpal patella (3 in *Charinus jibaossu* and 5 in *Charinus ruschii*), the size of the stalk of the female gonopod (long in *Charinus jibaossu* and short in *Charinus ruschii*) and the presence/absence of a constriction close to the apical border of the female gonopod (absent in *Charinus jibaossu* and present in *Charinus ruschii*). All specimens of *Charinus ruschii* have three spines on the pedipalp tarsus, whereas *Charinus jibaossu* is polymorphic, with one to three spines in this segment of the pedipalp (Vasconcelos et al. 2014).

*Charinus potiguar* is also similar to *Charinus ruschii*, but the frontal process is shorter, the dorsal pedipalp femur has 3 spines (in contrast to 5), the dorsal patella has 5 spines (in opposition to 6), the ventral patella has 2 spines (while the new species has 5), the pedipalp tarsus has 2 spines (*Charinus ruschii* has 3), the female has long stalked gonopod with divergent “V” shaped openings, and the proximal tooth of the basal segment of the chelicerae lacks the distal expansion.

The presence of well-developed median eyes and tubercle distinguish *Charinus ruschii* from *Charinus eleonorae* (which has reduced median eyes and tubercle) and from *Charinus troglobious* (which is completely blind). *Charinus eleonorae* also has a uniquely high number of setae on the frontal border of the carapace (ten) while all other species of the group have at most six. *Charinus troglobius* has a distinctively short tritosternum, which does not extend between the pedipalp coxae, a morphology that is quite different from almost all other species of the genus.

The pedipalp spine number is the distinguishing character between *Charinus ruschii* and *Charinus bromeliaea*. This species has three dorsal and three ventral spines on the femur, and four dorsal and two ventral patella spines. On the other hand, the femur of *Charinus ruschii* has five dorsal and five ventral, and the patella has six dorsal and five ventral spines. Moreover, *Charinus bromeliaea* has two dorsal spines on the pedipalpal tarsus, while *Charinus ruschii* has three. Similarly, *Charinus asturius* and *Charinus acaraje* have fewer spines on the pedipalp compared to *Charinus ruschii*. *Charinus acaraje* also possesses an extremely reduced frontal process, a character present only in this species.

*Charinus montanus* and *Charinus brasilianus* live in sympatry with *Charinus ruschii* (Fig. 4), but these species have striking differing characters. *Charinus montanus* is a unusual species due to the presence of clavate setae over most of the body (prosoma, legs and pedipalps), an acute projection in the frontal carapace border (anterior to the median eyes), tarsus I with 28 segments, extremely long first tarsus I segment, flattened and wide sternum platelets, and dark brown body color (even in old museum specimens). *Charinus brasilianus*, on the other hand, has the usual *Charinus* shape, but is also dark brown colored, and the female gonopod has a unique invagination on the frontal side. Moreover, *Charinus montanus* and *Charinus brasilianus* have seven teeth in the cheliceral claw, while *Charinus ruschii* has ten, and the two older species are considerably smaller than the new one (with the exception of the long pedipalps of the sexually dimorphic males of *Charinus brasilianus*; Table 2).

Regarding the other South American species of *Charinus*, the new species differs from *Charinus insularis*, *Charinus koepckei* and *Charinus vulgaris* by the absence of claws in the female genitalia, a feature present in the latter three species. *Charinus ruschii* is also different from *Charinus insularis* in the number of ventral spines on the femur (five in the new species and 3–4 in *Charinus insularis*), the number of articles in the tarsus I (41 in *Charinus ruschii* and 43 in *Charinus insularis*) and number of teeth in the cheliceral claw (ten in *Charinus ruschii* and 6–8 in *Charinus insularis*). *Charinus koepckei* differs from *Charinus ruschii* in the presence of the tetra and pentasternum formed by two separate small rounded concave platelets, trichobothria *bc* midway between *bf* and *sbf*, and the cheliceral claw with seven teeth. The Amazonian *Charinus vulgaris* has fewer spines on the pedipalp (e.g. 2–3 dorsal and three ventral on the femur), lacks the median eyes and tubercle, and has only four teeth on the cheliceral claw.

*Charinus ruschii* has similar appearance to *Charinus gertschi* Goodnight & Goodnight, 1946, but can be differentiated by the number of spines on dorsal patella (six in the former and five in the latter) and the shape of the sternum (small convex sclerotized platelets, in contrast to broad flatten platelets, respectively).

The four-articled basitibia IV differentiates *Charinus ruschii* from *Charinus quinteroi* Weygoldt, 2002 and *Charinus platnicki* (Quintero, 1986), which have just two, and from *Charinus bordoni* (Ravelo, 1975), *Charinus camachoi* (González-Sponga, 1998), *Charinus longitarsus* Armas & Palomino-Cárdenas (2016), *Charinus pardillalensis* and *Charinus tronchonii* (Ravelo, 1975), which have three. Besides that, *Charinus platnicki* has cushion-like gonopods and extremely reduced median eyes and tubercle; *Charinus tronchonii*, *Charinus bordoni*, *Charinus pardillalensis* and *Charinus camachoi* lack median eyes and tubercle; and *Charinus longitarsus* have a remarkable long first tarsal segment on leg I, which separates it from *Charinus ruschii*.

The other *Charinus* of the world can be distinguished by the shape of the female genitalia. The *australianus* species group has cushion-like gonopods, the *bengalensis* species group have finger-like gonopods and the *seychellarum* species do not have a gonopod.

This is one of the first detailed descriptions of leg I of a species of Charinidae. The number and amount of sensory structures is considerably different from that described for Phrynidae (Foelix et al. 1975, Igelmund 1987), but it is rather similar to that of *Charinus ioanniticus* (Kritscher, 1959), a distant related species (Miranda et al. in press). *Charinus ruschii* has fewer types of hairs and sensory openings than *Heterophrynus elaphus* Pocock, 1903, which has at least five different sensory structures (bristle sensilla, porous sensilla, club sensilla, rod sensilla, and the pit organ; Igelmund (1987)). *Charinus ruschii* has only two different sense organs: bristle sensilla and club sensilla (Fig. 3B–F). Another difference is the relative size of the claws at the end of tarsus I. *Charinus ruschii* has the lateral claws smaller than the median claw, while species of *Heterophrynus* have the opposite size relation (Foelix et al. (1975); Fig. 3E). The claws lack a subterminal opening, suggesting that in *Charinus* species the claws probably lack a sensory function, different to what is found in Phrynidae (Igelmund 1987).

### Behavior

The observations presented here enforce the “sit-and-wait” hunting strategy pattern within whip spiders (e.g. Alexander (1962), Gray and Robinson (1986), Pinto-da-Rocha et al. (2002), Weygoldt (2000)) and other arachnids (Mineo et al. 2003, Punzo 2000), with a frequent representation of immobility in their activity patterns.

More accurate studies are necessary to a complete understanding of survival tactics in Amblypygi, where comparisons between feeding strategies as well as activity rhythm in juveniles and adult males and females should be accessed. *Charinus ruschii* is currently known only from the type locality, Estação Biológica Santa Lúcia, and dwells in specific microhabitats, which might make them an endangered or vulnerable species in account to indiscriminate anthropic deforestation of the Atlantic Forest. The threat to this environment is of high concern due to the high levels of endemism (Myers et al. 2000).

## Supplementary Material

XML Treatment for Charinus ruschii
